# 

**Published:** 1872-07

**Authors:** 


### NOTICES.

Pure Farm-House Milk. — In warm weather it is of the utmost consequence that the milk fed to young children should be pure and fresh. In these degenerate days, there is one man at least who has acquired a handsome competence by persevering integrity under many discouragements — Samuel W. Cartfield, who is his own " middle-man." The milk is delivered to him at his own home in Orange County; it is his custom to come on the same train with his milk; it is delivered at his own offices, 411 Seventh Avenue, 71 Fourth Avenue and 1331 Broadway, where some of the members of the family are always in attendance in such a way that adulteration or mixture is impossible. We have used his milk a dozen years, and have always found it rich, creamy, and pure.

"The To-morrow of Death, or the Future Life According to Science;" by LOUIS FIGUIER, author of "Primitive Man," "Earth and Sea," etc. 395 pp., 12mo, published by Roberts Brothers, Boston, Mass. There are twenty-three chapters, the idea running through all is, What does science teach us about death? Man consists of soul, body, and the life. What is death? What becomes of these after death? Where does the superhuman being dwell? Do all become superhuman beings? The reincarnation of the wicked and infants. Subjects, all of which interest every intelligent man and woman.

MICROSCOPES. — Among the cheapest and most entertaining, is that sent post-paid for $3.00, by John Hall, Bergen, New Jersey.

The American Tract Society has issued "The Temptation in the Desert," being lessons from Christ's conflict and victory, by Rev. A. F. DIXON, 144 pp. 35 cts. Also, "Eve and Bertie," a tale for little children, by the author of "Hungering and Thirsting," "Willie and Lucy at the Sea-side," etc. 168 pp. 50 cts. Also for 30 cts. 152 pages, "The Old Paths," by Mrs. H. C. McKNIGHT. Twelve chapters on the "New Life," "Choosing," "Prayer," "Common Things," "Sabbath," "Giving," "Amusements," "Reading," etc.

"The Mental Cure," $1.50; 364 pp. 12mo, published by William White, Boston. The author, Rev. W. F. EVANS, gives facts illustrating the influence of the mind on the body, both in health and disease, proposing what he denominates the psychological method of treatment, meaning the cure of disease through the mind, one mind acting on another mind. The author is said to be able to cure patients by sitting down beside them, operating upon them by agencies going out from his mind to theirs. A great many curious facts are stated, and theories broached, and speculations indulged in. His neighbors at Claremont, New Hampshire, say many good and flattering things of him. If he had kept on his own side of the fence, and spent the whole force of his mind and heart in the cure of souls, and left us doctors to ourselves, to cultivate our own little truck patches to earn a decent living, it would have seemed more generous; but he is adding field to field like the balance of us. But it is better to cure the soul than the body, and all good people, and all wise men, regret at any time to see an efficient clergyman go at something else; there are too few such already; and those who do step aside, for any purpose whatever, generally come out at the little end of the horn. The Master does not like it, and He sends blasting, and blight, and mildew after them, very often, and when He does not, it's in mercy.

---

ORDERS for the JOURNAL, and everything pertaining to the business department, specimen numbers, and advertisements, should be addressed to Hurd and Houghton, 13 Astor Place, New York; The Riverside Press, Cambridge, Mass.

